# Factors associated with high cardiovascular risk in Putumayo

**DOI:** 10.15649/cuidarte.4207

**Published:** 2025-04-22

**Authors:** Jessica Paola Ruiz-Sandoval, Dayana Vizcaino-Sulbarán, Juan Pablo Álzate-Granados, Diana Isabel Cáceres-Rivera, Luis Alberto López-Romero

**Affiliations:** 1 Enfermera, Magister en epidemiología clínica. Fundación Universitaria De Ciencias de la Salud-FUCS. Bogotá, Colombia. jrsandoval9618@gmail.com Fundación Universitaria De Ciencias de la Salud-FUCS. Fundación Universitaria De Ciencias de la Salud-FUCS. Bogotá Colombia jrsandoval9618@gmail.com; 2 Especialista en epidemiología clínica Fundación Universitaria De Ciencias de la Salud-FUCS, Médico Universidad del Norte Colombia. Bogotá, Colombia. daya9606@gmail.com Fundación Universitaria De Ciencias de la Salud-FUCS. Universidad del Norte Colombia. Fundación Universitaria De Ciencias de la Salud-FUCS. Universidad del Norte Colombia. Bogotá Colombia daya9606@gmail.com; 3 Profesor asociado Fundación Universitaria de Ciencias de la Salud - Médico epidemiólogo. Candidato a doctorado en oncología. Bogotá, Colombia. jpalzate@fucsalud.edu.co Fundación Universitaria de Ciencias de la Salud Fundación Universitaria de Ciencias de la Salud Bogotá Colombia jpalzate@fucsalud.edu.co; 4 Enfermera, Magíster en Enfermería, PHD en Biomedicina. Universidad Cooperativa de Colombia. Bucaramanga, Colombia. dianai.caceres@ucc.edu.co Universidad Cooperativa de Colombia. Universidad Cooperativa de Colombia. Bucaramanga Colombia dianai.caceres@ucc.edu.co; 5 Enfermero, Magister en Epidemiología. Docente en la Escuela de Enfermería de la Universidad Industrial de Santander. Estudiante Doctorado en Metodología de la Investigación Biomédica y Salud Pública, Universidad Autónoma de Barcelona. Bucaramanga, Colombia. alberlop60@gmail.com Escuela de Enfermería de la Universidad Industrial de Santander. Universidad Autónoma de Barcelona. Escuela de Enfermería de la Universidad Industrial de Santander. Universidad Autónoma de Barcelona. Bucaramanga Colombia alberlop60@gmail.com

**Keywords:** Sociodemographic Factors, Health, Hypertension, Diabetes Mellitus, Cardiovascular Risk, Factores Sociodemográficos, Salud, Hipertensión, Diabetes Mellitus, Riesgo Cardiovascular, Fatores Sociodemográficos, Saúde, Hipertensão, Diabetes Mellitus, Risco Cardiovascular

## Abstract

**Introduction:**

Sociodemographic factors such as geographic location are associated with high cardiovascular risk. Urban areas are characterized by densely populated areas with access to services, while rural areas have fewer people and limited services.

**Objective:**

To determine the factors associated with high cardiovascular risk according to the area of patients enrolled in a chronic care program in Putumayo.

**Materials and Methods:**

Analytical cross-sectional study. The population was selected through simple random sampling. Information on the main risk factors was collected and included in a multivariate logistic regression model.

**Results:**

A total of 1,190 patients were included (median age 59.5 years [IQR 53–66], 68.74% women). 86.22% of the patients had high cardiovascular risk, with a similar distribution between urban (87.95%, n=628) and rural (83.61%, n=398) areas and a statistically significant difference compared to low/moderate risk (p=0.033). The factors related to cardiovascular risk in this population were primary education (OR: 0.68, CI 95%: 0.38–1.24), secondary education (OR: 0.88, CI 95%: 0.42–1.83), higher education (OR: 0.33, CI 95%: 0.13–0.82), ethnicity (none) (OR: 2.13, CI 95%: 0.98–4.63), rural area (OR: 0.66, CI 95%: 0.47–0.94), and contributory health affiliation (OR: 6.58, CI 95%: 2.75–15.72).

**Discussion:**

This study revealed that factors such as education level, ethnicity, type of health affiliation, and area were related to cardiovascular risk.

**Conclusion:**

The results showed a high proportion of individuals with elevated cardiovascular risk in Putumayo, with statistically significant differences between areas.

## Resumen

**Introducción:**

Existen factores sociodemográficos asociados al alto riesgo cardiovascular como la ubicación geográfica. La zona urbana se caracteriza por áreas densamente pobladas con acceso a servicios, mientras que la zona rural tiene menos población y servicios limitados.

**Objetivo:**

Determinar los factores asociados al riesgo cardiovascular alto según la zona en pacientes atendidos en un programa de crónicos en el Putumayo.

**Materiales y métodos:**

Estudio transversal analítico. La población se seleccionó a través de un muestreo aleatorio simple. Se recolectó la información de los principales factores de riesgo y estos se incluyeron en un modelo de regresión logística multivariada.

**Resultados:**

Se incluyeron 1.190 pacientes (mediana edad 59,5 años [RIC de 53–66], 68,74% mujeres). El 86,22% de los pacientes tenían riesgo cardiovascular alto, con una distribución similar entre zona urbana (87,95%, n=628) y rural (83,61%, n=398) y una diferencia estadísticamente significativa con el riesgo bajo/moderado (p=0.033). Los factores relacionados con el riesgo cardiovascular para esta población fueron la educación primaria con un OR: 0.68 IC 95%: 0,38 – 1,24, educación secundaria (OR: 0.88 IC 95%: 0,42–1,83), educación superior (OR: 0.33 IC 95%: 0,13- 0,82), etnia (ninguno) OR: 2.13 IC 95%: 0,98 – 4,63, zona (rural) OR: 0.66 IC 95%: 0,47- 0,94 y tipo de afiliación contributiva (OR: 6.58 IC 95%: 2,75- 15,72).

**Discusión:**

Este estudio reveló que factores como nivel educativo, etnia, tipo de afiliación y zona estuvieron relacionados con riesgo cardiovascular.

**Conclusiones:**

A partir de los resultados se evidenció una alta proporción de personas con riesgo cardiovascular elevado en el Putumayo, con diferencias estadísticamente significativas entre zona.

## Resumo

**Introdução:**

Existem fatores sociodemográficos associados ao alto risco cardiovascular, como a localização geográfica. A área urbana é caracterizada por áreas densamente povoadas com acesso a serviços, enquanto a área rural tem menos população e serviços limitados.

**Objetivo:**

Determinar os fatores associados ao alto risco cardiovascular de acordo com a área em pacientes tratados em um programa crônico em Putumayo.

**Materiais e métodos:**

Estudo transversal analítico. A população foi selecionada por amostragem aleatória simples. Informações sobre os principais fatores de risco foram coletadas e incluídas em uma regressão logística multivariada.

**Resultados:**

Foram incluídos 1190 pacientes (idade média de 59,5 anos [IQR 53–66], 68,74% mulheres). 86,22% dos pacientes apresentaram alto risco cardiovascular, com distribuição semelhante entre áreas urbanas (87,95%, n=628) e rurais (83,61%, n=398) e diferença estatisticamente significativa com risco baixo/moderado (p=0,033). Os fatores relacionados ao risco cardiovascular para essa população foram educação primária com OR: 0,68 IC 95%: 0,38 – 1,24, educação secundária (OR: 0,88 IC 95%: 0,42–1,83), educação superior (OR: 0,33 IC 95%: 0,13- 0,82), etnia (nenhuma) OR: 2,13 IC 95%: 0,98 – 4,63, área (rural) OR: 0,66 IC 95%:0,47- 0,94 e tipo de afiliação contributiva (OR: 6,58 IC 95%: 2,75- 15,72).

**Discussão:**

Este estudo revelou que fatores como nível educacional, etnia, tipo de afiliação e área estavam relacionados ao risco cardiovascular.

**Conclusões:**

Os resultados mostraram uma alta proporção de pessoas com risco cardiovascular elevado no Putumayo, com diferenças estatisticamente significativas entre as áreas.

## Introduction

 Cardiovascular risk (CVR) refers to the individual's probability of developing cardiovascular disease (CVD). It involves the occurrence of events such as myocardial infarction, cerebral hemorrhage, and embolism^1^, usually caused by obstructions that restrict blood flow to the heart or brain. These obstructions, in turn, result from the formation of atherosclerotic plaque or fatty deposits in the walls of blood vessels^2^.

 When talking about CVR, it is important to recognize that arterial hypertension (AHT) and type 2 diabetes mellitus (DM2) are the most relevant risk factors for cardiovascular events. In Latin America, the prevalence of hypertension is reported to range between 30% and 50%, while DM2 ranges between 8% and 13%^3^. In the department of Putumayo in the year 2020, the crude incidence rates per 100,000 inhabitants were 1.79 for AHT (n=354), 0.99 (n=354) for DM2, and 2.86 (n=1019) for chronic kidney disease, taking into account that mortality rates per 100,000 inhabitants for these conditions were 93.14 (n=332), 33.10 (n=118), and 46.01 (n=164), respectively^4^.

 Likewise, a frequently used tool recognized worldwide for measuring CVR is available: the Framingham scale, which estimates the 10-year risk in persons over 30 years. It classifies them as low risk if the sum of the scores is below 10%, moderate risk if it falls between 10% and 20%, and high risk if it is >20%^5^. In Colombia, this scale has been adapted by multiplying the obtained score by 0.75^6^. This tool becomes crucial in programs for patients with chronic conditions, as it helps identify CVR in a population at higher risk of developing cardiovascular disease (CVD) and requiring hospitalization due to their baseline condition, leading to an increased need for medical care^7^.

 On the other hand, despite Colombia’s high-coverage health system, barriers to healthcare access persist, particularly for those living in rural areas of the country^8^,^9^. The literature reports that the health situation in rural areas is more complex and is influenced by factors such as nutritional imbalances, high fertility rates, excessive workloads, poor environmental conditions^10^, and difficult access to education^11^. Consequently, these factors underscore the relevance of sociodemographic conditions in determining CVR, alongside the previously mentioned clinical factors.

 Therefore, assessing potential differences in CVR between rural and urban areas is important, considering the different sociodemographic and clinical characteristics. This assessment allowed us to answer the following question: What factors are associated with high cardiovascular risk in patients from rural areas compared to those residing in urban areas who are enrolled in a chronic disease program at health institutions in Putumayo?

## Materials and Methods

**Study design and population:** A cross-sectional study with an analytical component. The study population comprised adult patients enrolled in a chronic conditions program in one of the health service provider institutions (IPS, for its acronym in Spanish) in the department of Putumayo during the second semester of 2023. 

**Eligibility criteria:** Patients aged 30 to 74 years were included. Pregnant women and individuals with incomplete Framingham equation variables in the database, preventing cardiovascular risk calculation, were excluded. 

**Sample size and sampling:** The sample size was calculated for a logistic regression using the formula 




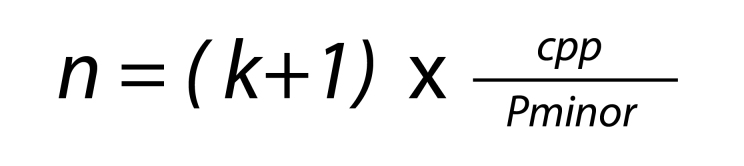




where n represents the required sample size, k is the number of variables included in the model (6 in this case), which was multiplied by 10 cases per variable, and finally divided by the prevalence of cardiovascular disease (the dependent variable) reported as 5.88%^12^. Based on these parameters, the estimated sample size was 1,190 patients. 

The sample was selected through simple random sampling, from a database provided by two participating IPS from urban low- and medium-complexity care centers, with an eligible population of 6,377 in the two municipalities. No missing values were obtained in the sample data. 

**Statistical analysis:** The analysis was conducted using Stata version 18 with the total sample (1,190). The geographic area of residence (rural or urban), educational level, ethnicity, type of health affiliation, cigarette smoking, history of AHT, history of DM, presence of dyslipidemia, and use of antihypertensive treatment were described using absolute and relative frequencies. According to the Framingham scale, age and CVR scores were reported as medians and interquartile ranges (IQR) after assessing normality with the Shapiro-Wilk test. 

CVR analyses were performed considering two categories according to the Framingham scale score: low/moderate and high. First, a comparison between the proportion of these CVR categories and area of residence (urban versus rural) was made using a χ2 test. Subsequently, bivariate analyses were conducted using logistic regression, considering CVR levels and each independent variable. Variables with a p≤0.25 in the bivariate analysis, along with those deemed clinically relevant, were included in a multivariate logistic regression model. For variables that were not statistically significant (educational level and ethnicity), the power calculations were performed, yielding 100% for both; consequently, for this sample, these variables were not associated. 

A multivariate logistic regression was conducted, confirming that all variables were significant for inclusion in the multivariate model. Subsequently, possible confounding variables were assessed using the coefficient change method, and interactions were examined by incorporating interaction terms into the logistic regression model. Finally, regression assumptions and model diagnostics were evaluated using the Hosmer-Lemeshow test, which yielded a p-value of 0.96, indicating a good model fit^13^. The complete dataset collected is freely accessible for consultation on Mendeley Data^14^. 

The study protocol was submitted for approval to the Ethics Committee of the Fundación Universitaria de Ciencias de la Salud (FUCS) and classified as “no-risk research”^15^ according to Resolution 8430 of 1993 issued by the Colombian Ministry of Health. Additionally, to safeguard privacy and confidentiality, subjects identified during sample construction were assigned sequentially ordered codes in compliance with the guidelines established in Law 1581 of 2012 to protect personal data^16^. In addition, this study was approved by the Research Ethics Committee of the Universidad Cooperativa de Colombia as per Minute No. 6, dated 06/22/2023. 

## Results

Results from 1,190 patients identified during the study period are presented. The median age of the study population was 59.5 years (IQR 53-66), with a median of 59 years in urban areas (IQR 52-66) and 60 years in rural areas (IQR 53-65). Regarding sex, a lower proportion of women was observed in urban areas (67.09%, n=479) compared to rural areas (71.22%, n=339). In terms of educational level, most of the population completed primary education, with a slight numerical difference between urban (66.95%, n=478) and rural areas (72.27%, n=344). Most patients had subsidized health affiliation in both urban (97.76%, n=698) and rural areas (98.11%, n=467). In addition, a comparison of variables and area (rural or urban) revealed statistically significant differences in educational level and ethnicity. Other demographic characteristics are shown in Table 1. 

**Table 1:** Sociodemographic variables of the study population

Sociodemographic variable	Total n = 1,190 % (n)	Rural n=476 % (n)	Urban n=714 % (n)	p-value**
Median age [IQR]	59.5 [53-66]	60 [53-65]	59 [52-66]	0.3432
Sex				0.132
Female	68.74 (818)	71.22 (339)	67.09 (479)	
Male	31.26 (372)	28.78 (137)	32.91 (235)	
Educational level				0.001
Illiterate	12.35 (147)	13.03 (62)	11.90 (85)	
Primary	69.08 (822)	72.27 (344)	66.95 (478)	
Secondary	14.87 (177)	11.97 (57)	16.81 (120)	
Technicians/University/Other	3.70 (44)	2.73 (13)	4.34 (31)	
Ethnic group				0.003
Ethnic minority	3.28 (39)	14 (2,94)	25 (3.50)	
None	96.72 (1151)	97.06 (462)	96.50 (689)	
Type of health affiliation				0.680
Subsidized	97.90 (1165)	98.11 (467)	97.76 (698)	
Contributory	2.10 (25)	1.89 (9)	2.24 (16)	

*IQR: Interquartile range; ** χ2 test for categorical variables and Mann-Whitney U test.

Regarding the study population's risk factors and clinical conditions, the proportion of tobacco dependence is low in urban (1.96%, n=14) and rural areas (1.47%, n=7). The proportion of dyslipidemia was similar in both areas (urban 35.15%, n=251 and rural 36.76%, n=175). Type II diabetes was proportionally more frequent in urban areas (64.99%, n=464) than in rural areas (67.65%, n=322). The AHT proportion was high in both urban (91.32%, n=652) and rural (89.50%, n=426) areas. In addition, a comparison of clinical variables by area (rural or urban) revealed statistically significant differences in diastolic blood pressure, antihypertensive treatment, glycosylated hemoglobin, total cholesterol, and LDL cholesterol. Other clinical characteristics are shown in Table 2. 

**Table 2:** Clinical variables

Clinical variables	Total n = 1,190 % (n)	Rural n = 476 % (n)	Urban n = 714 % (n)	p-value
Smoker	1.76 (21)	1.47 (7)	1.96 (14)	0.529
Dyslipidemia	35.80 (426)	36.76 (175)	35.15 (251)	0.570
Diabetes Mellitus				0.307
Type I	24.12 (287)	21.85 (104)	25.63 (183)	
Type II	66.05 (786)	67.65 (322)	64.99 (464)	
No diabetes	9.83 (117)	10.50 (50)	9.38 (67)	
Arterial hypertension	90.59 (1078)	89.50 (426)	91.32 (652)	0.393
Systolic blood pressure				
Median [IQR]	130 [120-144]	130 [120-140]	130 [ 120-146]	0.184
Diastolic blood pressure				
Median [IQR]	80 [80-90]	80 [80-90]	80 [80-90]	0.026
Antihypertensive Treatment				0.002
Non-pharmacological management	3.28 (39)	5.04 (24)	2.10 (15)	
HCTZ	1.60 (19)	1.68 (8)	1.54 (11)	
ACE inhibitor or ARA	21.60 (257)	22.27 (106)	21.15 (151)	
HCTZ + ACE inhibitor or ARA	22.77 (271)	21.01 (100)	23.95 (171)	
HCTZ + ACE inhibitor or ARA + Amlodipine	23.36 (278)	18.07 (86)	26.89 (192)	
Amlodipine	1.01 (12)	1.47 (7)	0.70 (5)	
HCTZ + ACE inhibitor or ARA + Amlodipine + Other	12.94 (154)	15.97 (76)	10.92 (78)	
Other	0.92 (11)	0.84 (4)	0.98 (7)	
No information	12.52 (149)	13.66 (65)	11.76 (84)	
Glycated Hemoglobin				0.003
Normal (<5.7)	40.08 (477)	45.17 (215)	36.69 (262)	
Prediabetes (5.7 – 6.4)	4.79 (57)	3.99 (19)	5.32 (38)	
Diabetes (>6.4)	55.13 (656)	50.84 (242)	57.98 (414)	
Blood glucose				0.497
Normal (<99)	46.13 (530)	47.59 (217)	45.17 (313)	
Prediabetes (100-125)	30.55 (351)	30.04 (137)	30.88 (214)	
Diabetes (>126)	23.32 (268)	22.37 (102)	23.95 (166)	
Total cholesterol				0.000
Normal (<200 mg/dL)	59.66 (710)	55.04 (262)	62.75 (448)	
Normal – High (200 - 240 mg/dL)	25.55 (304)	26.89 (128)	24.65 (176)	
High (>240 mg/dL)	14.79 (176)	18.07 (86)	12.61 (90)	
HDL				0.177
Normal	79.50 (946)	78.36 (373)	80.25 (573)	
Low	20.50 (244)	21.64 (103)	19.75 (141)	
LDL				0.000
Optimal (<100)	46.10 (544)	40.80 (193)	49.65 (351)	
Near optimal (100-130)	27.88 (329)	27.48 (130)	28.15 (199)	
Upper limit of the normal range (>130 – 160)	16.69 (197)	19.24 (91)	14.99 (106)	
High (>160 – 189)	6.53 (77)	8.03 (38)	5.52 (39)	
Very high (>189)	2.80 (33)	4.44 (21)	1.70 (12)	
Triglycerides				0.146
Normal (<150)	39.83 (474)	42.02 (200)	38.38 (274)	
Borderline high (150 – 200)	19.24 (229)	18.91 (90)	19.47 (139)	
High (>200)	40.92 (487)	39.08 (186)	42.16 (301)	

*IQR: Interquartile range; HCTZ: Hydrochlorothiazide; ACE: Angiotensin-Converting Enzyme; ARA: Angiotensin Receptor Antagonists. HDL: High-Density Lipoprotein LDL: Low-Density Lipoprotein

**Figure 1:**
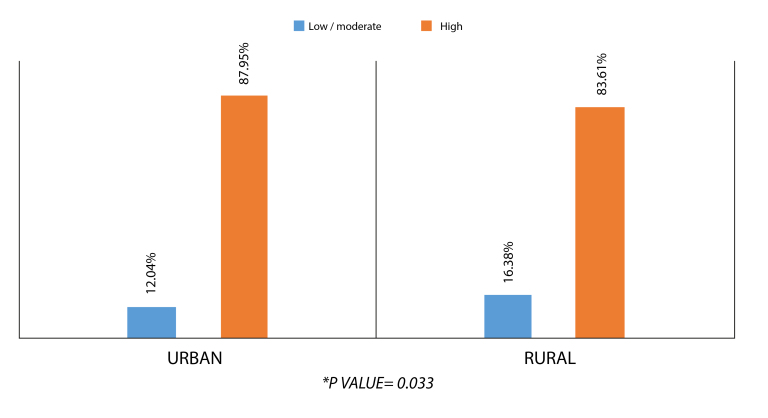
Cardiovascular risk in patients living in rural areas vs. population living in urban areas

The most frequent CVR level for the study population was high, with a numerically higher proportion in the urban areas (87.95%) compared to the rural areas (83.61%). When comparing CVR level with geographic areas of residence, a statistically significant difference was found (p=0.033) (See Figures 1).

* χ2 test with a p-value = 0.033.

Finally, within the results observed in the multiple logistic regression, the final model included the following variables: primary education (OR: 0.68, CI 95%: 0.38–1.24), secondary education (OR: 0.88, CI 95%: 0.42–1.83), higher education (OR: 0.33, CI 95%: 0.13–0.82), rural area (OR: 0.66, CI 95%: 0.47–0.94), subsidized health affiliation (OR: 6.58, CI 95%: 2.75–15.72), and ethnicity (none) (OR: 2.13, CI 95%: 0.98–4.63). The latter did not reach statistical significance (see Table 3). Confounding and interaction variables were assessed, and none were identified. 

**Table 3:** Sociodemographic factors associated with cardiovascular risk (Framingham*)

Variable	Unadjusted OR (95%CI)	p-value	Adjusted OR** of the multivariate model (95% CI)	p-value
Education level				
Illiterate	1		1	
Primary	0.64 (0.36 –1.16)	0.145	0.68 (0.38 -1.24)	0.216
Secondary	0.82 (0.40 –1.69)	0.604	0.88 (0.42 -1.83)	0.736
Technicians/University/Other	0.20 (0.08 –0.46)	0.000	0.33 (0.13-0.82)	0.017
Ethnic group				
Ethnic minority	1		1	
None	1.92 (0.89 – 4.13)	0.092	2.13 (0.98- 4.63)	0.054
Area				
Urban	1		1	
Rural	0.69 (0.50 – 0.97)	0.034	0.66 (0.47-0.94)	0.021
Type of health affiliation				
Contributory	1		1	
Subsidized	8.61 (3.83-19.32)	0.000	6.58 (2.75- 15.72)	0.000

* Output/Framingham Variable: Age, total cholesterol, HDL, systolic blood pressure, smoking, and diabetes mellitus; **Multivariate odds ratios adjusted for other variables.

## Discussion

This study's results suggest that there is a statistically significant difference in cardiovascular risk levels among patients enrolled in a chronic condition care program in the department of Putumayo, depending on their geographic location. This finding aligns with a 2013 study^17^ conducted in an urban population, which reported high CVR in 98.7% of the study population. However, it contrasts with the rural population findings of that study, where only 2.3% exhibited high CVR, a numerical difference compared to the 83.61% identified in this study. The high proportion of cardiovascular risk observed in Putumayo may be attributed to the fact that individuals who visit these IPS health centers are patients with symptoms or a history of cardiovascular risk-related conditions, as opposed to healthy individuals who also access healthcare services. 

We found that educational level, area, and type of health affiliation were associated with higher cardiovascular risk. In the final logistic regression model, educational level was statistically significant in the technical/university/other category, indicating that the possibility of having a higher level of education among patients with high cardiovascular risk is one-third that of patients who are not at high cardiovascular risk. This finding is consistent with a study published in April 2019, which related low educational levels to higher cardiovascular mortality and suggested that individuals with higher education are more knowledgeable about the disease, its risk factors, and preventive measures^5^,^18^. 

Subsidized health affiliation was identified as a risk factor for developing severe cardiovascular problems compared to contributory health affiliation. As observed in previous studies, no statistically or clinically significant association was found between cardiovascular risk and type of health affiliation^19^. However, according to a recent study, individuals with a subsidized health affiliation exhibited better glycemic index control than those with a contributory health affiliation^20^. Given that diabetes and hypertension contribute to or increase CVR, glycemic control can be considered an indirect indicator of cardiovascular risk. According to the healthcare system's functioning, high cardiovascular risk levels are expected to be distributed independently of the type of health affiliation. However, in this study sample, the high proportion of individuals with subsidized health affiliation may indicate a possible association. This finding could also be explained by selection bias. 

Ethnicity in the final logistic regression model was not statistically significant, with an OR of 2.13 and a 95% CI of 0.98–4.63. However, the literature shows that ethnicity is a predisposing factor for cardiovascular disease. A document published by the Spanish Heart Society cites multiple studies identifying a greater predisposition to AHT among individuals of black ethnicity, with evidence suggesting a worse prognosis in this population^21^,^22^. However, in Colombia, there is a mixture of cultures and races; therefore, these characteristics are measured by official entities using patient self-reporting, which poses challenges at the moment of objectively classifying them for data analysis. Additionally, for this analysis, the ethnicity variable was analyzed by grouping individuals at risk —Romanies, Gypsies, Black, and Afro-Colombian populations—compared to White individuals. 

This study made it possible to know cardiovascular risk levels among patients attending chronic care units and living in rural and urban areas, revealing statistically significant differences between these areas in the department of Putumayo. These findings, in turn, provide the basis for formulating hypotheses in future research, as the study offers an overview of potential relationships between the variables of interest. 

It is important to mention that the main limitation of this study is the apparent selection bias, as the sample consisted of patients enrolled in chronic care programs. This is why these individuals are more likely to have risk factors, which could lead to overestimating cardiovascular risk. It is relevant to recognize that not all patients with risk factors fall into the high-risk category. Therefore, conducting risk classifications during patient follow-up is important for improving the accuracy of cardiovascular risk assessment, particularly for subclinical cardiovascular disease, silent ischemia, and future cardiovascular events. Additionally, risk classifications could prevent the unnecessary use of treatments in low-risk patients, thereby increasing adverse events^23^. It should also be mentioned that these programs' recruitment rates of chronic patients do not reach the entire population with CVR. However, an attempt was made to mitigate this bias by selecting participants through simple random sampling. 

he possibility of measurement bias cannot be ruled out, as the main source of information was medical records, which in these municipalities are transcribed manually. Additionally, misclassification bias may have occurred, as errors in the variables used in the equation for calculating cardiovascular risk could have led to a change in the categorization of patients (low, moderate, or high risk). Consequently, such misclassification of information could also impact the interpretation of the results. 

The importance of cardiovascular risk assessment and the identification of risk factors has long been a subject of research. Although many risk-enhancing factors have been identified, it is necessary to determine whether this risk is the same in different populations. Current health patterns, urbanization, dietary changes, access to healthcare services, and medical care may also influence the development of cardiovascular disease. This will enable the identification of potential socioeconomic factors to be considered in populations with specific characteristics, allowing the implementation of long-term, sustainable strategies to improve health outcomes. In turn, this could help mitigate the rising prevalence of cardiovascular diseases and reduce health costs^18^. 

**Implications for practice**

The implications for clinical practice of this study lie in its potential to enhance the accuracy of cardiovascular risk classification and, consequently, optimize medical care. The findings of this study reveal significant differences in cardiovascular risk between rural and urban areas of Putumayo, highlighting the importance of adapting prevention and treatment strategies to the specific characteristics of each population. In clinical practice, these insights would allow health professionals to implement more personalized interventions, tailored to patients' socioeconomic and geographic conditions. Ultimately, this study establishes a starting point for future research and changes in clinical practice that will allow more efficient management of cardiovascular diseases, contributing to better public health and reducing the associated healthcare costs. 

## Conclusions

This study determined that there is a high proportion of individuals with elevated cardiovascular risk in Putumayo, with statistically significant differences between rural and urban areas. We declare that, due to selection bias, the population with which the statistical analyses were performed is not representative of the entire population of Putumayo. Therefore, the data can only be extrapolated to populations with chronic conditions. Cardiovascular risk was associated with educational level, ethnicity, type of health affiliation, and area.
